# Toward improving photosynthesis in cassava: Characterizing photosynthetic limitations in four current African cultivars

**DOI:** 10.1002/fes3.130

**Published:** 2018-04-16

**Authors:** Amanda P. De Souza, Stephen P. Long

**Affiliations:** ^1^ Departments of Crop Sciences and Plant Biology Carl R Woese Institute for Genomic Biology University of Illinois at Urbana‐Champaign Urbana IL USA; ^2^ Lancaster Environment Centre Lancaster University Lancaster UK

**Keywords:** carbon assimilation, food security, genetic engineering, sub‐Saharan Africa, yield improvement

## Abstract

Despite the vast importance of cassava (*Manihot esculenta* Crantz) for smallholder farmers in Africa, yields per unit land area have not increased over the past 55 years. Genetic engineering or breeding for increased photosynthetic efficiency may represent a new approach. This requires the understanding of limitations to photosynthesis within existing germplasm. Here, leaf photosynthetic gas exchange, leaf carbon and nitrogen content, and nonstructural carbohydrates content and growth were analyzed in four high‐yielding and farm‐preferred African cultivars: two landraces (TME 7, TME 419) and two improved lines (TMS 98/0581 and TMS 30572). Surprisingly, the two landraces had, on average, 18% higher light‐saturating leaf CO_2_ uptake (*A*_sat_) than the improved lines due to higher maximum apparent carboxylation rates of Rubisco carboxylation (*V*_cmax_) and regeneration of ribulose‐1,5‐biphosphate expressed as electron transport rate (*J*_max_). TME 419 also showed a greater intrinsic water use efficiency. Except for the cultivar TMS 30572, photosynthesis in cassava showed a triose phosphate utilization (TPU) limitation at high intercellular [CO_2_]. The capacity for TPU in the leaf would not limit photosynthesis rates under current conditions, but without modification would be a barrier to increasing photosynthetic efficiency to levels predicted possible in this crop. The lower capacity of the lines improved through breeding, may perhaps reflect the predominant need, until now, in cassava breeding for improved disease and pest resistance. However, the availability today of equipment for high‐throughput screening of photosynthetic capacity provides a means to select for maintenance or improvement of photosynthetic capacity while also selecting for pest and disease resistance.

## 1 INTRODUCTION

Cassava (*Manihot esculenta* Crantz) is the third largest source of calories in tropical and subtropical regions after rice and maize (FAO, 2008) and considered a staple food for more than a billion people in 105 countries (Chetty, Rossin, Gruissem, Vanderschuren, & Rey, 2013). Additionally, cassava is a primary source of income for smallholder farmers in Africa (Nweke, 2005). Despite significant efforts in breeding and agronomy, cassava productivity in sub‐Saharan Africa has declined at a rate of 0.024 t ha^−1^ year^−1^ between 2004 and 2014. In Nigeria, the world's largest producer, yields per unit land area have barely increased over the past 55 years (De Souza et al., 2017).

A recent review has suggested that large increases in cassava yields might be achieved by bioengineering or molecular breeding of increased photosynthetic efficiency given that the observed efficiency is only ~14% of the theoretical values for a C_3_ photosynthesis (De Souza et al., 2017). Although there are instances where increased photosynthesis at the level of individual leaves did not correspond to increased biomass accumulation (Long, Zhu, Naidu, & Ort, 2006), positive relationships between leaf photosynthesis and productivity have been demonstrated in cassava in a range of experiments (El‐Sharkawy, 2016). Critically, when grown under open‐air CO_2_ elevation as a means to artificially increase net photosynthetic efficiency by inhibiting photorespiration, a very large increase in tuber yield was observed (Rosenthal et al., 2012). One of the keys to succeeding in increasing crop photosynthesis is to understand the possible limitations of photosynthesis. While rates and limitations are well characterized for photosynthesis in crops of importance to the temperate zone, such as maize, rice, wheat, and soybean, little is known of these properties in crops limited to the tropics—in particular, those of importance in Sub‐Saharan Africa (De Souza et al., 2017; Long, Marshall‐Colon, & Zhu, 2015). Studies of photosynthesis in cassava are scarce and mainly for South American cultivars. However, it is in Africa where cassava is of greatest importance to smallholder farmers.

The efficiency with which a leaf can capture incident light and use it to assimilate carbon defines leaf photosynthetic efficiency under light‐limiting conditions. Such efficiency is determined by the apparent maximum quantum yield of CO_2_ assimilation (ɸCO_2_) that is described as the initial slope of the photosynthetic response to photosynthetic photon flux density (Long, Farage, & Garcia, 1996). Under nonlimiting light conditions, however, limitation to C_3_ photosynthesis can be due to stomatal limitation of CO_2_ uptake and limitations within the mesophyll. Within the mesophyll limitation can be both by transfer of CO_2_ from the intercellular space to the site of carboxylation within the chloroplast (mesophyll conductance; *g*_m_) and biochemical limitation at carboxylation. The latter is controlled by the minimum value of the maximum rate of ribulose 1,5‐bisphosphate carboxylase/oxygenase (Rubisco) catalyzed carboxylation (*V*_cmax_; Rubisco limited), the regeneration of ribulose 1,5‐bisphosphate (RuBP) controlled by electron transport rate (*J*_max_; RuBP limited), and less frequently, with the rate of inorganic phosphate released from the utilization of triose phosphates (*V*_TPU_; TPU or P_i_ limited) (Farquhar, Von Caemmerer, & Berry, 1980; Long & Bernacchi, 2003) . All three may be determined from *A*/*c*_i_ curves, that is, the fitted response of light‐saturated leaf CO_2_ uptake (*A*_sat_) to a range of intercellular CO_2_ concentrations (*c*_i_). Stomatal limitation may also be quantified from *A*/*c*_i_ curves (Farquhar & Sharkey, 1982).

Limited sink capacity is known to feedback the photosynthetic process, reducing the photosynthetic rates due to an accumulation of starch in leaves (Stitt, 1991). In cassava, although tuberous roots function as a large sink of carbohydrates throughout the development, the lack of relationship between canopy photosynthesis and biomass production in some cultivars suggests that there are cassava varieties that might be sink limited (El‐Sharkawy & De Tafur, 2007; Ihemere, Arias‐Garzon, Lawrence, & Sayre, 2006; Pellet & El‐Sharkawy, 1994). Establishment, that is, the early growth of the crop from planting to formation of a closed leaf canopy, is a key stage in the production of any crop. Leaf photosynthesis at this stage is critical since it determines the rate of supply of carbohydrates to fuel the development of the leaf canopy to capture more light and, in turn, provide for photosynthate as well as for the establishment of the root system. This exponential phase of growth is also when sink limitation should be least.

Here, we evaluate the factors that limit photosynthesis in cassava in the establishment phase by using four high‐yield and farm‐preferred African cultivars. Within this, we compare two landraces with two bred cultivars, to assess possible impacts of breeding selection.

## 2 MATERIALS AND METHODS

### 2.1 Plant material and experimental conditions

Four cassava (*M. esculenta*, Crantz) cultivars (TME 7, TME 419, TMS 98/0581 and TMS 30572) considered high yielding and with high popularity among farmers in Nigeria (Agwu, Njom, & Umeh, 2017; Oriola & Raji, 2013) were used in this study. The cultivars TME 7 (also called “Oko‐Iwayo”) and TME 419 are African landraces, whereas TMS 98/0581 and TMS 30572 are improved cultivars bred by the International Institute of Tropical Agriculture (IITA).

Photosynthetic gas exchange, leaf carbon and nitrogen contents, dimension of leaf anatomical features, and growth of these four cultivars were measured in two independent experiments. These experiments included six biological replicates of each cultivar in a completely randomized design. The experiments were conducted in a controlled environment greenhouse from 27 May to 13 July and from 1 July to 18 August 2016 at the University of Illinois at Urbana–Champaign. To assess possible sink limitation, a third experiment with five biological replicates was performed during 19 August and 29 September of the same year. This third experiment comprised measurement of the diurnal course of gas exchange and nonstructural carbohydrate contents, as detailed below.

For all three experiments, the four cassava cultivars were propagated in vitro as described in Bull et al. (2009). Individual stem cuttings of ~ 1.5 cm were placed in sterile 53 mm × 100 mm plastic jars with solid cassava basic culture medium (4.4% Murashige and Skoog medium with vitamins, 20% sucrose, 0.1% 2 mmol/L CuSO_4_, and 2.5% gelrite, pH 5.8 ; Bull et al., 2009). The transparent containers were maintained in a walk‐in growth chamber with 16/8 hr light/dark, at 26°C. After 5–7 days, the stem cuttings sprouted and generated a new plantlet. After 30 days, plantlets of similar sizes were gently pulled from the medium, the roots were washed in lukewarm tap water to remove the excess of medium, and the plantlets were transferred to 0.7 L pots containing soil mix (LC1 Sunshine mix; Sun Gro Horticulture, Agawam, MA, USA). The leaves were sprayed with water to keep the foliage moist, and transparent plastic domes were placed over the pots inside the greenhouse to maintain a high (<90%) air humidity. After 3 days, the domes were gradually opened to allow the plants to acclimate to the air humidity inside the greenhouse. Fourteen days after plantlet transference (DAT) to the greenhouse, they were transferred to 14.4 L pots containing soil mix (LC1 Sunshine mix; Sun Gro Horticulture, Agawam, MA, USA) and fertilized with 30 g of NPK 15:9:12. Pots were distributed inside the greenhouse according to a fully randomized design with 25 cm spacing and rotated every 3 days to avoid confounding environmental variation within the greenhouse with individual plants and cultivars. Plants were watered to field capacity every 2–3 days.

Air temperature and relative humidity within the greenhouse were measured using a combined temperature–humidity sensor (HMP60‐L; Vaisala Oyj, Helsinki, Finland) and light intensity using a quantum sensor (LI‐190R; LI‐COR, Lincoln, NE, USA), and all continuously recorded (Figure S1; CR1000; Campbell Scientific Inc., Logan, UT, USA).

### 2.2 Leaf gas exchanges measurements

The responses of leaf CO_2_ uptake rate (*A*) to photosynthetic photon flux density (PPFD) (*A*/PPFD curves) and to intracellular CO_2_ concentration (*c*_i_) (*A*/*c*_i_ curves) were determined for the youngest fully expanded leaf of each plant with a portable open gas‐exchange system coupled with a leaf chamber chlorophyll fluorometer and light source (LI‐6400XT and LI‐6400‐40; LI‐COR) in plants of 40–42 DAT from two independent experiments. For the *A*/PPFD curves, the reference CO_2_ concentration was maintained at 400 μmol mol^−1^. Plants were first induced to steady state at a PPFD of 1,800 μmol m^−2^ s^−1^, and then, the PPFD was decreased following the sequence: 1,500, 1,000, 800, 600, 400, 250, 150, 75, 50, 25, and 0 μmol m^−2 ^s^−1^. For the *A*/*c*_i_ curves, PPFD level was set to 1,800 μmol m^−2 ^s^−1^ and after a steady‐state *A* was obtained, the reference CO_2_ concentration was varied according to the sequence: 400, 270, 150, 100, 75, 50, 400, 400, 600, 800, 1,100, 1,300, and 1,600 μmol mol^−1^, following in the procedure of Long and Bernacchi (2003). For all the measurements, leaf temperature was maintained at 28 ± 1°C and a chamber water vapor pressure deficit (VPD) of 1.1–1.7 kPa.

The maximum apparent carboxylation rate by Rubisco (*V*_cmax_), the regeneration of ribulose‐1,5‐biphosphate expressed as electron transport rate (*J*_max_), and triose phosphate utilization (*V*_TPU_) were calculated from *A*/*c*_i_ curves using the equations from von Caemmerer (2000). As calculation of the true *V*_cmax_ requires the knowledge of the exact mesophyll conductance (*g*_m_) under each measurement condition, the term apparent is used to denote the fact that the given estimates of *V*_cmax_ reflect both *g*_m_ and the true *V*_cmax_. Before fitting the curves, values were corrected for diffusive leaks between the chamber and the surrounding atmosphere, based on measurements with an empty chamber. Values obtained at 28°C were adjusted for temperature response to 25°C according to Bernacchi, Singsass, Pimentel, Portis, and Long (2001) and McMurtrie and Wang (1993). Stomatal limitation (*l*_s_) was determined from the response of *A* to *c*_i_ as described by Long and Bernacchi (2003). Maximum apparent quantum yield (ɸCO_2_) and leaf respiration (*R*_d_) were calculated from fitting the *A*/PPFD curves to a nonrectangular hyperbola (Long & Hällgren, 1993). Light‐saturated leaf carbon assimilation (*A*_sat_) was considered as the value obtained at 1,800 μmol m^−2 ^s^−1^ PPFD. Stomatal conductance (*g*_s_) and intracellular CO_2_ concentration at 400 μmol mol^−1^ (*c*_i_) were calculated after von Caemmerer and Farquhar (1981) and intrinsic water use efficiency (iWUE) obtained by dividing *A* by *g*_s_.

To describe the diurnal course *A*,* g*_s_ and iWUE were determined every 2.5 hr from 40 min after dawn to 40 min before dusk (7:30 a.m., 10:00 a.m., 12:30 p.m., 3:00 p.m., and 5:30 p.m.) using a different set of plants to those used for the preceding determination of *A*/PPFD and *A*/*c*_i_ evaluation. The light intensity, relative air humidity, and leaf temperature inside the leaf chamber were set to ambient values measured inside the greenhouse before each time point.

### 2.3 Leaf cross section analysis

At 45 days after transplanting (DAT), leaf pieces of ~0.8 cm^2^ were cut from the middle of the central lobe of the most recently expanded leaf in eight replicate plants. They were immediately placed in 70% ethanol and then maintained under vacuum for 24 hr. Samples were then subjected to dehydration in an ethanol/butanol series (Johansen, 1940) and finally embedded in paraffin wax. Transverse sections of 5 μm were cut using a microtome (Leica RM 2125 RMS; Leica Biosystems, Buffalo Grove, IL, USA). Sections were mounted on glass slides and stained with 0.1% toluidine blue. Images were captured using a digital scanner system (NanoZoomer 2.0‐HT; Hamamatsu Photonics K.K., Bridgewater, NJ, USA). Palisade, spongy, and total leaf thickness were measured digitally using the ruler tool in the Nanozoomer Digital Pathology viewer (NDP.view ‐ version 2.6/Rev.1; Hamamatsu Photonics K.K.). For each biological replicate, palisade, spongy, and total leaf thickness were measured in two different leaf sections and in six different locations within each section, totaling 12 measurements per parameter per biological replicate; 96 in total per cultivar. These 12 values were averaged to comprise a value for each individual plant.

### 2.4 Growth measurements

The number of leaves, specific leaf area (SLA), total leaf area, stem height, average internode length, and biomass production were recorded at 45 DAT. The SLA for individual plants was calculated as the average from three leaf disks of 3.8 cm^2^ each, collected at midday. The disks were oven dried at 60°C for 48 hr and weighed.

Stem height was determined as the vertical distance from the root–shoot transition to the insertion of the newest leaf. In order to determine the average internode length, the stem height was divided by the number of leaves. At final harvest, soil was removed by washing and then each plant was divided into leaves, petioles, stem, and tuberous roots. These were oven dried at 60°C to constant weight. Total leaf area was calculated as the product of leaf biomass and SLA.

### 2.5 Leaf carbon and nitrogen content

Leaf disks used for SLA determination were then ground to a fine powder in a ball mill (Geno Grinder 2010, Lebanon, NJ, USA). Two milligrams of each sample were weighed into tin capsules, and the total carbon (C) and nitrogen (N) content were quantified using an elemental analyzer (Elemental Combustion System CHNS‐O, Costech ECS 4010, Valencia, CA, USA). Acetanilide and apple leaves (National Institute of Science and Technologies Inc., Valencia, CA, USA) were used as standards. The content of C and N was expressed in percentage of dry mass. Nitrogen use efficiency (NUE) was calculated as the ratio of leaf N and *A*_sat_.

### 2.6 Nonstructural carbohydrates

In the third experiment, central portions of (a) the middle lobe from the youngest fully expanded leaf, (b) the petiole carrying the youngest fully expanded leaf, (c) stem, and (d) tuberous root were sampled and immediately frozen into liquid nitrogen. Samples from five biological replicates per cultivar were collected at dusk (~30 min before the end of the photoperiod) and on the following dawn (~30 min before the beginning of the photoperiod).

Samples were freeze‐dried (Labconco Freezone 4.5 Freeze Dry System, Labconco, MO, USA) and then ground in a ball mill (Geno Grinder 2010, Lebanon, NJ, USA). To extract the soluble sugars, 10 mg of each sample was subjected to four 80% ethanolic extractions at 80°C as described by De Souza, Arundale, Dohleman, Long, and Buckeridge (2013). After each extraction, the supernatants obtained after centrifugation (10,000 *g*, 5 min) were combined, dried under vacuum (Savant SPD121P SpeedVac^®^, Thermo Fisher Scientific, MA, USA), and resuspended in 1 ml of ultrapurified water. Total soluble sugars (TSS) were quantified by the phenol‐sulfuric acid method (Dubois, Gilles, Hamilton, Rebers, & Smith, 1956) adapted for microplates (Masuko et al., 2005). High purity glucose (1 mg ml^−1^) was used as a standard.

The remaining pellets after the ethanolic extractions were oven dried at 40°C for 24 hr. The starch from these pellets was extracted enzymatically using α‐amylase (120 U ml^−1^) and amyloglucosidase (30 U ml^−1^) following De Souza et al. (2013). After incubation with a glucose oxidase/peroxidase assay kit (NZYtech, Lisboa, Portugal) at 30°C for 15 min, the glucose released from the enzymatic extractions was quantified spectrophotometrically at λ = 490 nm. Starch was calculated as being 90% of the total glucose released after enzymatic extraction (Amaral, Gaspar, Costa, Aidar, & Buckeridge, 2008).

### 2.7 Statistical analysis

The normality of each measured variables was tested using the Shapiro–Wilk's test and homogeneity of variance using Brown–Forsythe's and Levene's tests. When the data showed normal distribution and homoscedasticity, one‐way ANOVA followed by Tukey's test (*p *<* *.05) using cultivars as fixed factor was applied to separate means, where significance was indicated. In the absence of normal distribution or homoscedasticity, the data were transformed until normality was obtained. Where this was not possible, Wilcoxon's nonparametric comparison was used. Gas exchange, growth, leaf carbon and nitrogen content, and leaf thickness datasets from the two independent experiments were analyzed using a completely randomized block design with two blocks: *n *=* *8 for leaf C, leaf N, and leaf thickness and *n *=* *12 for gas exchanges and growth data (JMP^®^ Pro, version 12.0.1; SAS Institute Inc., Cary, NC, USA).

## 3 RESULTS

### 3.1 Leaf photosynthesis, [C], [N], and anatomy

**Figure 1:**
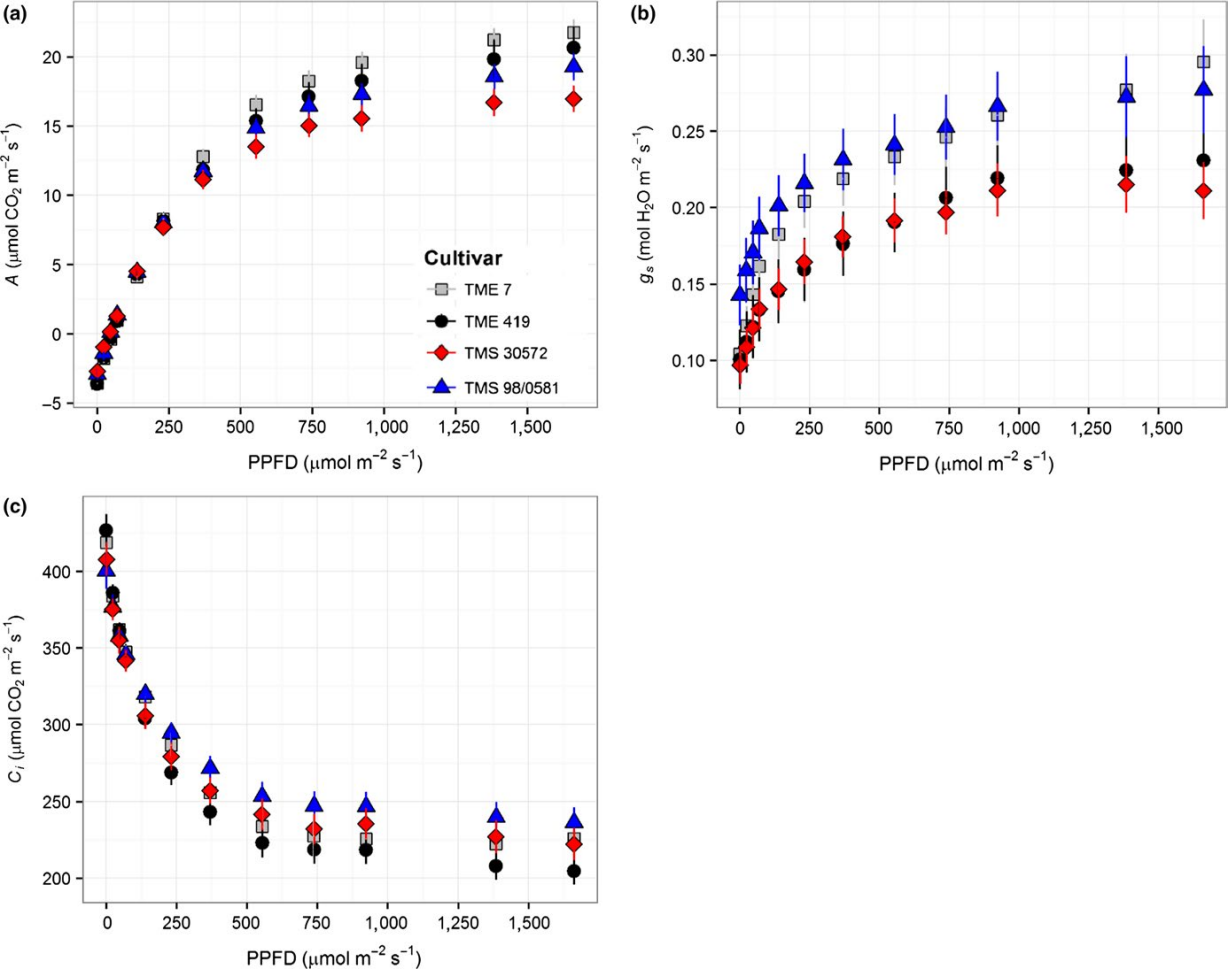
Responses of (a) net leaf CO_2_ uptake (*A*), (b), stomatal conductance (*g*_s_), and (c) intracellular CO_2_ concentration (*c*_i_) to photosynthetic photon flux density (PPFD) of four cassava cultivars during the establishment phase, at 40–42 days after transplanting of cloned plantlets. Symbols represent mean ± *SE*. *n *=* *12

**Figure 2:**
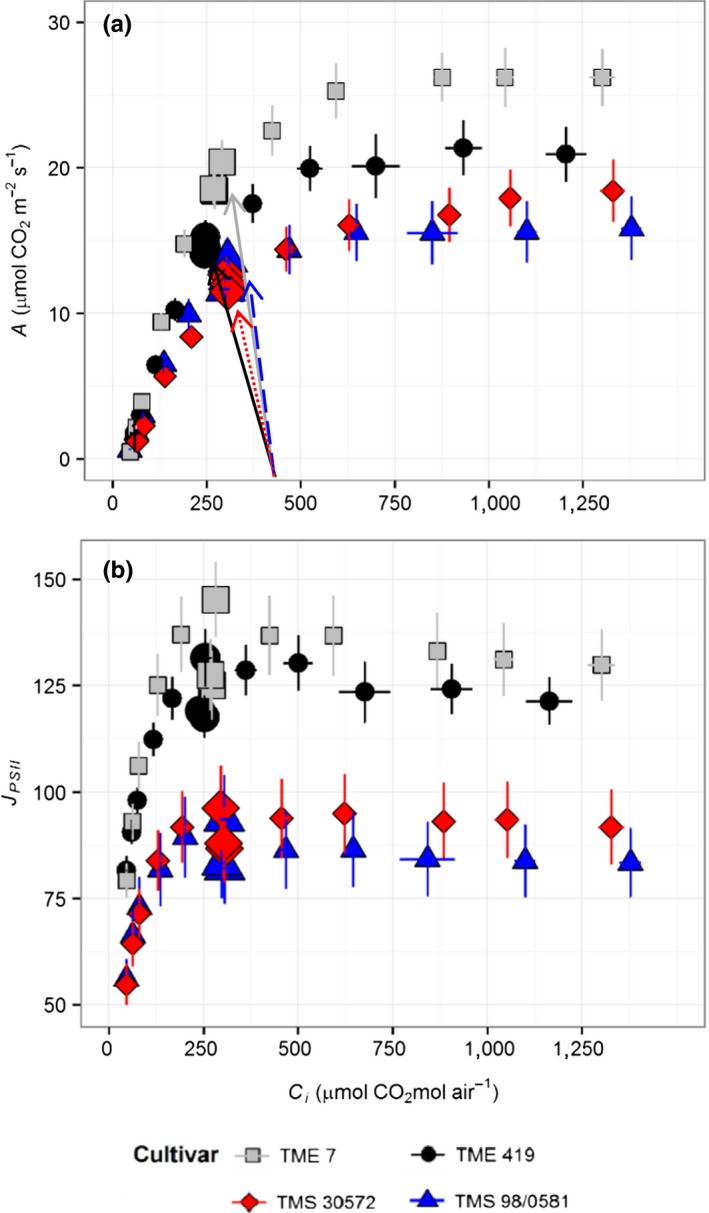
Responses of light‐saturated (a) net leaf CO_2_ uptake (*A*) and (b) electron transport rate (*J*_PSII_) to intracellular CO_2_ concentration (*c*_i_) for four cassava cultivars at 40–42 days after transplanting of cloned plantlets. Symbols represent mean ± *SE*. *n *=* *12. Larger symbols indicate the operating point and arrows indicate the supply function for each cultivar. The operating point is at the *c*_i_ achieved when the [CO_2_] around the measured leaf equals the current ambient level of 400 μmol mol^−1^

Leaf CO_2_ uptake rate (*A*) increased hyperbolically with light for all cultivars, with the two landraces showing higher light saturated rates (*A*_sat_) than the improved cultivars (Figure 1a). For irradiances higher than 600 μmol m^−2 ^s^−1^, TME 7 showed the highest *A*, followed by TME 419 with TMS 30572 having the lowest *A* (Figure 1a). The *A*_sat_ of ca. 23 μmol CO_2_ m^−2 ^s^−1^ from the *A*/PPFD curves for the two landraces was 18% greater (*p* = .0002) than in the bred cultivars (Table 1). This was tested by pooling the results for the two landraces and the two improved lines. No significant differences among the cultivars were observed in the apparent maximum quantum yield (ɸCO_2_), that is, the initial slope of the response of *A* to PPDF, or in leaf respiration (*R*_d_). Across light levels, stomatal conductance (*g*_s_) was, on average, 27% higher in TME 7 and TMS 98/0581 than in TME 419 and TMS 30572 (Figure 1b). This difference corresponded to a significant and substantial increase in intrinsic leaf water use efficiency (iWUE) in TME 419 and TMS 30572 (Table 1). Substantial differences in iWUE were also found between the two landraces. iWUE was 25% greater in TME 419 compared to TME 7, which was also reflected in a lower *c*_i_ at all PPFD (Figure 1C). Figure 2a shows that this higher iWUE was at the expense of a lower *A* in TME 419 at ambient [CO_2_] due to lower *g*_s_, as illustrated by the slope of the supply line.

**Table 1:** Maximum Rubisco‐catalyzed carboxylation rate (*V*_cmax_, μmol m^−2 ^s^−1^), regeneration of ribulose‐1,5‐bisphosphate represented by electron transport rate (*J*_max_, μmol m^−2 ^s^−1^), triose phosphate utilization rate (*V*_TPU_, μmol m^−2 ^s^−1^), light‐saturated leaf CO_2_ uptake (*A*_sat_, μmol CO_2_ m^−2 ^s^−1^), maximum apparent quantum yield of photosynthesis as a measure of light‐limited photosynthesis (ɸ, mol CO_2_ mol^−1 ^photon), leaf respiration (*R*_d_, μmol CO_2_ m^−2 ^s^−1^), stomatal limitation (*l*_s_), stomatal conductance (*g*_s_, mol H_2_O m^−2 ^s^−1^), intracellular CO_2_ concentration at 400 μmol mol^−1^ (*c*_i_, μmol CO_2_ m^−2 ^s^−1^), intrinsic water use efficiency (iWUE, μmol CO_2 _mol H_2_O^−1^), nitrogen use efficiency (NUE, μmol CO_2%_ leaf nitrogen^−1^), leaf carbon (C, % of dry weight) content, leaf nitrogen (N, % of dry weight) content, and leaf carbon:nitrogen ratio in the four cassava cultivars at 40–42 days after transplanting of cloned plantlets. n.d = not determined because there was no evidence that this cultivar was TPU limited at any *c*_i_

	Cultivar			
	TME 7	TME 419	TMS 30572	TMS 98/0581
*V*_cmax_	82.66 ± 6.93 A	71.06 ± 7.85 AB	44.65 ± 6.35 C	49.82 ± 5.96 BC
*J*_max_	133.17 ± 9.30 A	105.35 ± 10.54 A	70.35 ± 9.13 B	78.28 ± 8.91 B
*V*_TPU_	9.88 ± 2.85 A	9.16 ± 2.64 A	n.d.	6.81 ± 1.97 B
*A*_sat_	23.54 ± 0.98 A	22.78 ± 1.38 A	18.34 ± 1.01 B	19.61 ± 0.63 AB
ɸ	0.061 ± 0.004 A	0.064 ± 0.003 A	0.060 ± 0.008 A	0.061 ± 0.006 A
*R*_d_	3.53 ± 0.36 A	3.7 ± 0.39 A	2.77 ± 0.44 A	3.00 ± 0.46 A
*l*_s_	0.546 ± 0.019 B	0.646 ± 0.091 A	0.523 ± 0.027 B	0.521 ± 0.053 B
*g*_s_	0.32 ± 0.09 A	0.24 ± 0.10 A	0.24 ± 0.08 A	0.30 ± 0.10 A
*c*_i_	256.61 ± 8.58 A	227.77 ± 7.76 A	237.59 ± 13.24 A	256.13 ± 10.23 A
iWUE	74.13 ± 5.24 B	100.43 ± 4.51 A	83 ± 7.31 AB	78.25 ± 5.68 B
NUE	5.00 ± 0.63 A	5.38 ± 1.07 A	4.90 ± 0.92 A	4.71 ± 0.70 A
Leaf C	46.70 ± 0.09 A	46.72 ± 0.11 A	46.12 ± 0.11 B	46.05 ± 0.15 B
Leaf N	6.07 ± 0.06 A	5.46 ± 0.08 B	5.48 ± 0.11 B	5.78 ± 0.05 AB
Leaf C:N	7.70 ± 0.08 C	8.58 ± 0.13 A	8.40 ± 0.20 AB	7.97 ± 0.06 BC

Values represent mean ± *SE*. *n *=* *12 for gas exchange parameters; *n *=* *8 for leaf C, leaf N, and leaf C:N.

Different letters represent statistically significant differences (*p *<* *.05) among the cultivars

The higher *A* in TME 7 and TME 419 was noticeable at all *c*_i_ values (Figure 2a). The higher *A* in the two landraces was associated with a 35% and 28% greater apparent *V*_cmax_ and *J*_max_, respectively (Table 1). The term apparent is used here since *g*_*m*_ was not measured, and so differences may be in Rubisco activity, *g*_m_, or both. At *c*_i_ above 750 μmol mol^−1^, *A* showed no further increase indicating TPU limitation for TME 7, TME 419, and TMS 98/0581 (Figure 2a). This is confirmed by the fact that, in these cultivars, electron transport rate (*J*_PSII_) declines (Figure 2b). This reduction in *J*_PSII_ at high *c*_i_ was more accentuated in the two landraces TME 7 and TME 419 than in the improved line TMS 98/0581 and apparently absent in TMS 30572. Due to the absence of reduction in *J*_PSII_ at high *c*_i_ in TMS 30572, TPU limitation for this cultivar could not be calculated; the results implying that any TPU limitation in this cultivar could only occur about the higher CO_2_ concentration used and well above contemporary atmospheric levels. Although TME 419 had the second highest *A* at ambient [CO_2_], it showed the greatest stomatal limitation (*l*_s_), 22% higher than for the other cultivars, which is consistent with its higher iWUE (Table 1).

Even though leaf C content was significantly greater in the two landraces compared to the two improved cultivars, the difference was only 0.6% (Table 1). N content of TME 7 leaves was substantially higher than the other cultivars, corresponding to its greater RubP limited and RubP saturated *A* (Figure 2). However, no significant difference in leaf NUE was observed across the cultivars (Table 1).

**Figure 3:**
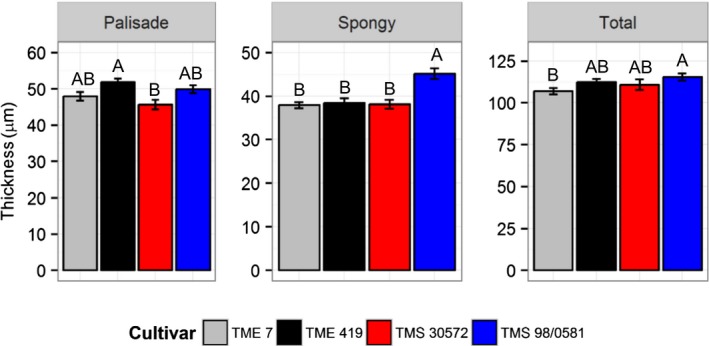
Palisade, spongy, and leaf thickness (μm) of the four cassava cultivars at 45 days after transplanting of cloned plantlets. Symbols are mean ± *SE*. Different letters represent statistically significant differences (*p *<* *.05) among the cultivars. Measurements were made on transverse sections of eight different leaves (*n *=* *8)

Higher photosynthetic rates per unit leaf area can often be associated with thicker leaves. However, here the cultivar with the thinnest leaves, TME 7, actually showed the highest *A* at all values of *c*_i_ (Figures 2 and 3). Leaf thickness was greatest in TMS 98/0581, mainly as a result of a thicker spongy mesophyll (Figure 3). The other cultivars were similar to each other in their leaf thickness.

### 3.2 Diurnal course of photosynthesis and nonstructural carbohydrates

**Figure 4:**
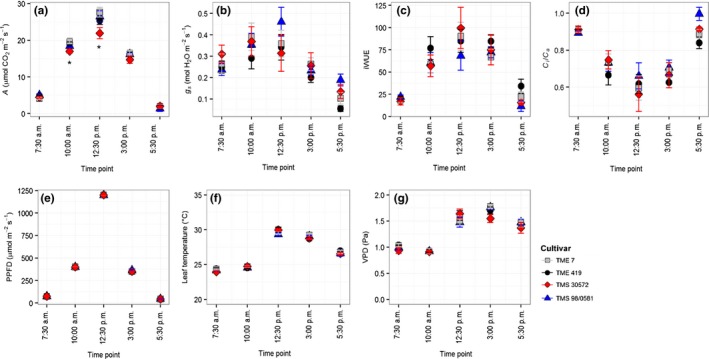
Diurnal course of (a) net leaf CO_2_ uptake (*A*), (b) stomatal conductance (*g*_*s*_), (c) intrinsic water use efficiency (iWUE), and (d) the ratio of intracellular to ambient CO_2_ concentration (*c*_i_/*c*_a_) of the four cassava cultivars at 40 days after transplanting of cloned plantlets. The lower panels show the diurnal course of (e) photosynthetic photon flux density (PPFD), (f) leaf temperature, and (g) water vapor pressure deficit (VPD). Symbols represent mean ± *SE*. *n *=* *5. Asterisks indicate statistically significant differences among cultivars at a given time point (*p *<* *.05)

The diurnal measurements of *A* showed the same pattern observed in the *A*/PPFD curves, with TMS 30572 values being the lowest at high light intensities during the day (Figure 4a). In agreement with the response to PPFD (Figure 1), *g*_s_ was not significantly different between cultivars. TMS 98/0581 appeared to have the highest *g*_s_, which was reflected in a poorer iWUE (Figure 4b,c). This was also consistent with the higher *c*_i_/*c*_a_ of this cultivar, which was particularly pronounced during the afternoon (Figure 4d).

**Figure 5:**
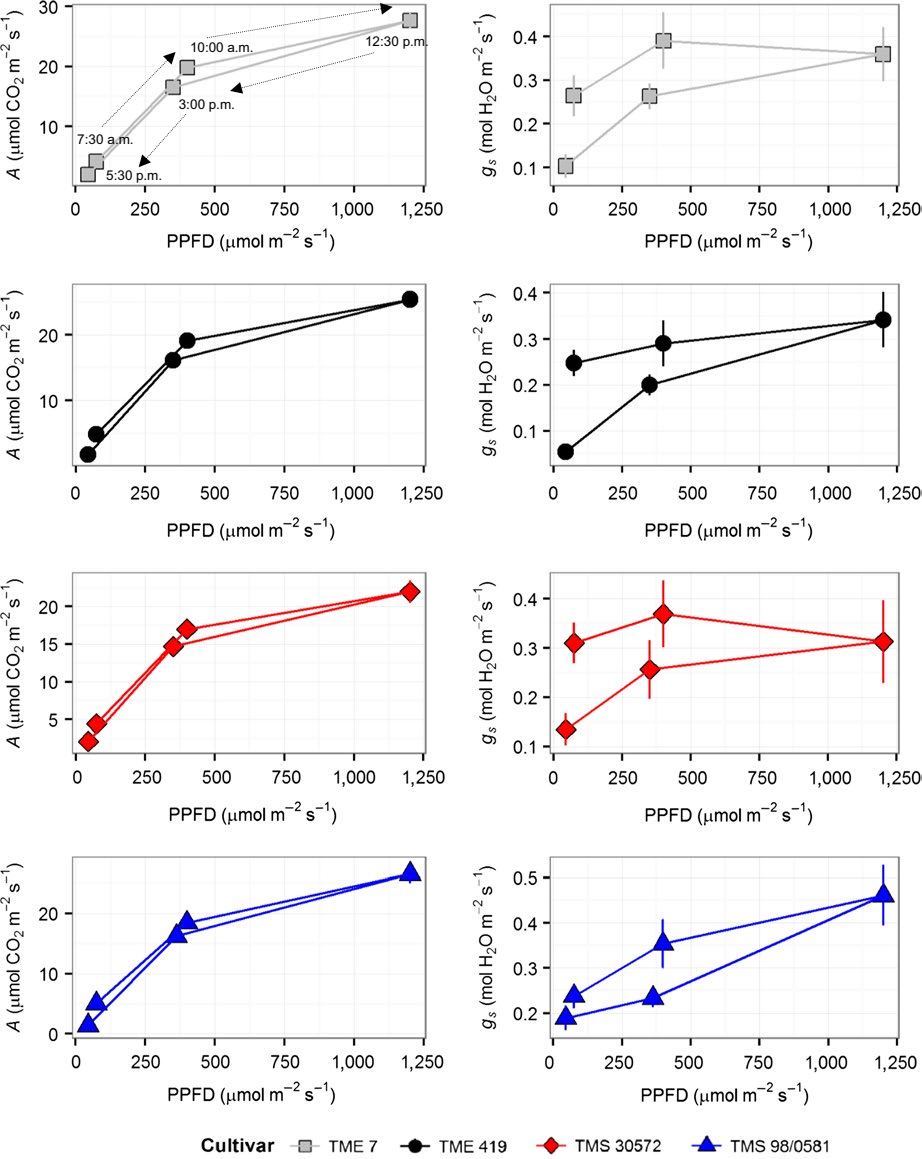
Progression of net leaf CO_2_ uptake (*A*) and stomatal conductance (*g*_s_) in response to photosynthetic photon flux density (PPFD) during a diurnal course measurements in the four cassava cultivars at 40 days after transplanting the cloned plantlets. Data was replotted from Figure 4. Arrows and time points indicated in the first panel are valid for all panels

The comparison of *A* between time points, where PPFD in the morning and afternoon were similar (i.e., 10:00 a.m. and 3:00 p.m.; 7:30 a.m. and 5:30 p.m.) (Figure 4e), did not show significant differences (Figure 5). Although there was evidence of hysteresis in the measured *A*, reductions in *g*_s_ by 20%–80% in the afternoon were found; the degree of reduction was varying with cultivar (Figure 5). The decrease in *g*_s_ during the afternoon corresponded to an increase of 0.5–0.7 Pa in water VPD between morning and afternoon (Figure 4g).

At dusk, leaf starch in TMS 30572 was lower than in the other cultivars (Table 2), possibly reflecting the lower *A* of this cultivar (Figures 1 and 4a). However, this cultivar showed a slightly higher starch accumulation in its tuberous roots, although no statistically significant differences were found between the cultivars (Table 2). Interestingly, TMS 30572 accumulated, proportionally, more starch in the tuberous roots than the other cultivars (Figure S2). The cultivar TMS 98/0581 had a larger starch accumulation in the stem (Table 2), and proportionally, accumulated more starch in this organ than in the tuberous roots (Figure S2).

**Table 2:** Starch and total soluble sugar (TSS) content (mg g^−1^) in leaf, petiole, stem, and tuberous root in the four cassava cultivars at dusk and at dawn at 40–42 days after transplanting the cloned plantlets to the greenhouse

	Cultivar	Starch		TSS	
		Dusk	Dawn	Dusk	Dawn
Leaf	TME 7	53.71 ± 18.21 ABa	9.33 ± 1.58 ABb	117.67 ± 4.31 Aa	121.09 ± 8.83 Aa
	TME 419	62.94 ± 4.8 Aa	11.73 ± 1.32 Ab	130.92 ± 8.38 Aa	127.79 ± 4.40 Aa
	TMS 30572	33.98 ± 4.31 Ba	3.93 ± 0.88 Bb	145.07 ± 11.49 Aa	97.94 ± 3.73 Bb
	TMS 98/0581	58.38 ± 5.5 Aa	4.13 ± 0.59 Bb	120.91 ± 5.64 Aa	124.08 ± 8.16 Aa
Petiole	TME 7	9.13 ± 0.84 Aa	12.78 ± 0.29 Aa	282.19 ± 37.50 Aa	344.89 ± 33.23ABa
	TME 419	7.99 ± 1.84 Aa	5.68 ± 0.53 Aa	323.64 ± 37.87 Aa	276.06 ± 14.66 Ba
	TMS 30572	7.1 ± 1.49 Aa	3.74 ± 0.69 Aa	359.70 ± 43.38 Aa	368.49 ± 31.20 Aa
	TMS 98/0581	7.01 ± 1.55 Aa	3.46 ± 0.57 Aa	310.65 ± 21.85 Aa	339.40 ± 13.79 ABa
Stem	TME 7	13.9 ± 2.48 ABa	8.17 ± 1.73 Aa	152.30 ± 14.95 Bb	257.62 ± 19.35 Aa
	TME 419	9.11 ± 0.86 Ba	4.41 ± 0.3 Ab	306.73 ± 31.93 Aa	277.74 ± 21.63 Aa
	TMS 30572	13.72 ± 1.6 Ba	6.46 ± 0.87 Ab	225.76 ± 14.42 Ab	326.88 ± 32.40 Aa
	TMS 98/0581	25.19 ± 3.58 Aa	7.48 ± 1.49 Ab	194.80 ± 19.41 ABa	233.04 ± 18.57 Aa
Tuberous root	TME 7	54.85 ± 5.53 Aa	60.01 ± 11.42 Aa	173.61 ± 13.17 Ba	207.86 ± 17.31 Ba
	TME 419	74.49 ± 14.79 Aa	56.53 ± 9.72 Aa	251.52 ± 7.97 Aa	297.90 ± 18.32 Aa
	TMS 30572	89.91 ± 14.82 Aa	53.77 ± 8.46 Aa	239.89 ± 16.34 Aa	264.08 ± 19.17 ABa
	TMS 98/0581	41.15 ± 8.75 Aa	39.63 ± 8.4 Aa	194.29 ± 27.90 ABb	282.75 ± 30.10 ABa

Values represent mean ± *SE*. *n *=* *5. Different letters represent statistically significant differences (*p *<* *.05).

Upper case letters indicate the comparison among the cultivars, and lower case letters indicate the comparison between dusk and dawn values for each cultivar.

Leaf starch was 80%–90% lower at dawn than at dusk for all cultivars. Except for the cultivar TME 7, stem starch at dawn reduced at 50%–70% of dusk of the preceding day (Table 2). Significant differences among the cultivars were found only in leaves, in which the cultivars TME 7 and TME 419 had higher starch content at the end of the night.

Total soluble sugar content in leaves and petioles at dusk did not vary among cultivars. In stem and tuberous roots, however, TSS at dusk in TME 419 and TMS 30572 was 30%–42.5% higher than in TME 7 (Table 2). Compared to dusk values, TMS 30572 showed a ~32% reduction in leaf TSS at dawn whereas the other cultivars maintained similar TSS contents in their leaves. In the stem, TSS accumulated between dusk and dawn in TME 7 (+69%) and TMS 30572 (+45%) while a TSS accumulation of 45% was observed in the tuberous roots of the cultivar TMS 98/0581 (Table 2).

### 3.3 Growth and biomass accumulation

**Figure 6:**
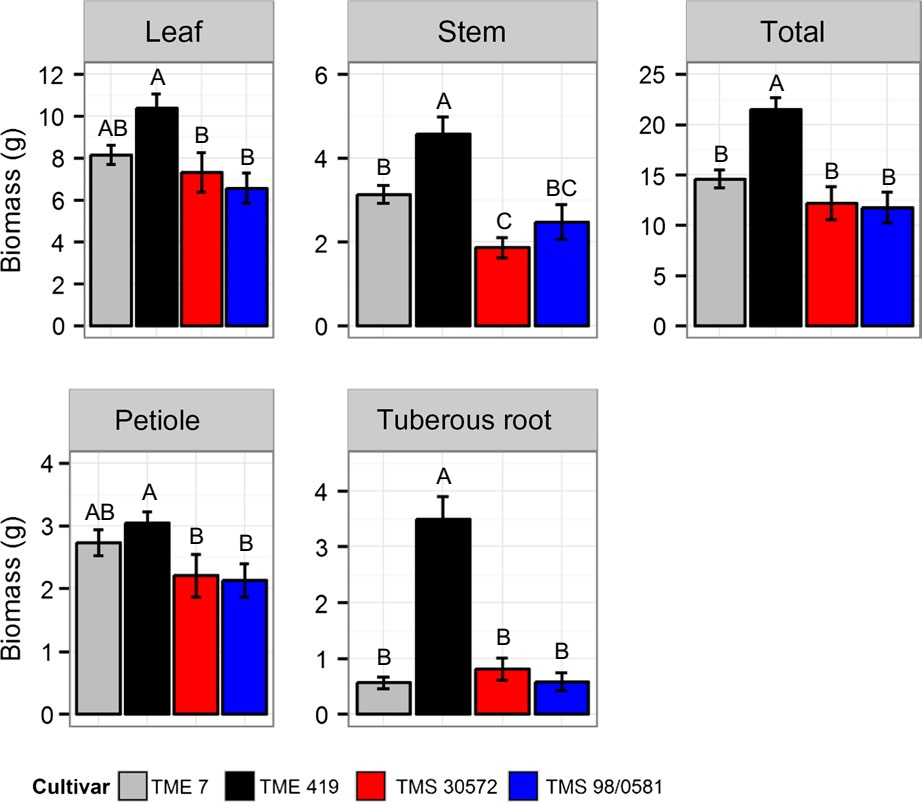
The dry weight of the whole plant, leaf, petiole, stem, and tuberous roots (g) of the four cassava cultivars at 45 days after transplanting of cloned plantlets. Bars represent mean ± *SE*. *n *=* *12. Different letters indicate statistically significant differences (*p *<* *.05) among the cultivars

After 45 days of growth, TME 419 showed the largest total biomass compared to the other three cultivars due to an 11% higher investment in leaves, 84% in stem, 29% in petioles, and 435% in tuberous roots (Figure 6). This cultivar also showed higher biomass partitioning to its tuberous roots (Figure S3). The other three cultivars showed similar total biomass (Figure 6), with small differences in the biomass partitioning among the organs (Figure S3). The cultivar TMS 30572 had lower stem biomass, shorter stems, and shorter internodes compared to the other cultivars (Figure 6, Table 3). Leaf number and leaf area did not differ significantly among the cultivars, although SLA in TME 419 was lower (Table 3).

**Table 3:** Number of leaves, leaf area (m^2^), specific leaf area (SLA, cm^2 ^mg^−1^), stem height (cm), and internode length (cm) in the four cassava cultivars at 45 days after transplanting the cloned plantlets to the greenhouse

	Cultivar			
	TME 7	TME 419	TMS 30572	TMS 98/0581
Number of leaves	17.92 ± 0.89 A	19.67 ± 0.66 A	17.67 ± 1.15 A	16.25 ± 1.07 A
Leaf area	0.316 ± 0.016 A	0.349 ± 0.014 A	0.292 ± 0.035 A	0.267 ± 0.022 A
SLA	0.39 ± 0.01 A	0.34 ± 0.01 B	0.42 ± 0.01 A	0.43 ± 0.01 A
Stem height	48.83 ± 1.96 AB	56.76 ± 3.30 A	40.15 ± 2.64 B	44.31 ± 3.38 B
Internode length	2.91 ± 0.09 A	2.91 ± 0.17 A	2.29 ± 0.08 B	2.73 ± 0.08 A

Values represent mean ± *SE*. *n *=* *12.

Different letters represent statistically significant differences (*p *<* *.05) among the cultivars.

## 4 DISCUSSION

Four African cassava cultivars considered high‐yielding and farmer‐preferred were evaluated to identify factors that can limit photosynthesis under steady state during the establishment phase of this crop. This revealed the maximum apparent quantum yield of CO_2_ uptake (ϕCO_2_) to be high and not significantly different among the cultivars, ranging from 0.060 to 0.064 (Table 1). Ehleringer and Pearcy (1983) recorded the values of 0.047–0.055 across a range of C_3_ species measured at 30°C and a measurement [CO_2_] of 330 μmol mol^−1^. This would be fully consistent with the values measured here at the higher measurement [CO_2_] of 400 μmol mol^−1^ and slightly lower measurement temperature of 28°C, both differences lowering photorespiration and increasing ϕCO_2_. These high values suggest that, at steady state in shade, photosynthetic rates are close to the theoretical maximum. By contrast, there was considerable variation in light‐saturated leaf CO_2_ uptake (*A*_sat_) at ambient and varied [CO_2_] conditions (Figure 2). We assessed aspects of photosynthetic limitation to *A*_sat_ in vivo related to inferred Rubisco activity, RuBP regeneration, triose phosphate utilization, stomatal conductance, and sink capacity. The findings suggest that, during this important crop establishment, phase limitations to light‐saturated photosynthesis are related to constraints in apparent *V*_cmax_, *J*_max_, and *g*_s_ and in some instances sink, depending on the cultivar. The variability found and the limitations identified indicate breeding and bioengineering strategies that could improve photosynthetic efficiency in this key crop. The surprisingly low *V*_TPU_ suggests a need to create greater sink capacity within the leaf for starch and sucrose synthesis. This limitation is likely to slow the all‐important early growth and establishment of the crop. High rates at this growth stage allow faster expansion of the canopy and increase crop photosynthesis and faster development of the root system, critical in protecting the crop against water shortages later in the life of the crop.

Curiously, when the *A*_sat_ values for the two landraces and the two improved lines were pooled together, landraces showed higher rates of *A*_sat_ than the improved cultivars. The same trend was observed at the operating point from *A*/c_*i*_ curves (Figure 2a), although these values were slightly lower than determined in the light response curves. This might be explained by the fact that these measurements were taken on different days and not necessarily on the same plants. The operating *c*_i_ at ambient atmospheric [CO_2_] in all cultivars was at the transition between RubP saturated and RubP limited conditions (Figure 2). The higher *A*_sat_ of the landraces was, therefore, a result of both higher *J*_max_ and apparent *V*_cmax_ (Figures 1a and 2a; Table 1). Mesophyll conductance (*g*_m_) was not measured in this study, but could also contribute to the higher apparent *V*_cmax_ and *J*_max_. Typically, high *V*_cmax_ values are positively correlated with leaf N (Walker et al., 2014). Consistent with that expectation, TME 7 showed the highest leaf N content and the highest apparent *V*_cmax_ (Table 1). On the other hand, the improved line TMS 30572 appeared to be Rubisco and RuBP regeneration limited given the low values of *V*_cmax_ and *J*_max_ (Figures 1a and 2a; Table 1). *V*_cmax_ as measured here is proportional to the total concentration of enzyme sites and *g*_m_ (von Caemmerer, 2000). Thus, lower values of *V*_cmax_ can be associated with lower amounts of Rubisco, lower Rubisco activation, lower *g*_m_, and/or within species variation in kinetic properties of Rubisco (von Caemmerer, 2000). In C_3_ plants, Rubisco comprises about 50% of the total soluble leaf nitrogen. Therefore, significant changes in the amount of Rubisco are likely to be reflected in the leaf N content. In this case, our results suggest that the level *V*_cmax_ observed in TMS 30572 may be related to reduced Rubisco activity or mesophyll conductance instead of reduced Rubisco content, since the leaf N in TMS 30572 was similar to the cultivar TME 419, which had has significantly higher *A*_sat_ and *V*_cmax_ than TMS 30572 (Table 1). This may reflect the wide variation in Rubisco activity found previously across a range of cassava germplasm (El‐Sharkawy, 2004, 2006).

*A*_sat_ at ambient [CO_2_] in TME 419 and TME 7 did not differ significantly (Figure 1a; Table 1). However, the stomatal conductance in TME 419 was slightly lower (Figure 1b) leading to a significantly greater stomatal limitation which allowed a higher intrinsic leaf water use efficiency while achieving a similar rate of leaf CO_2_ uptake (Table 1). In regions with frequent or extended drought periods such as many of those where cassava is grown, reduced *g*_*s*_ and improved iWUE are desirable traits (Sinclair & Muchow, 2001). Nonetheless, when the reduction in *g*_s_ is high enough to limit photosynthesis, it can also restrict the carbon uptake during nonstress conditions. Despite the high‐photosynthetic rates of TME 419, it is possible to suggest that the photosynthetic capacity of this cultivar can be even higher if *l*_s_ was reduced.

Although the SLA values observed in this study are higher than usually expected for cassava (Table 3), it is close to the range observed by Pujol, Salager, Beltran, Bousquet, and McKey (2008) in 6‐month‐old cassava plants. SLA in the cultivar TME 419 was lower compared to the other three cultivars (Table 3) even the thickness of its leaves was similar to both TMS 30572 and TMS 98/0581 (Figure 3). This result suggests that TME 419 has a more compacted leaf, with a smaller intracellular air space volume. Plants with lower SLA and lower intracellular air spaces tend to have lower *g*_*m*_ than plants with high SLA (Walker et al., 2014; Xiong, Flexas, Yu, Peng, & Huang, 2017).

While *A*_sat_ values of the two landraces TME 7 and TME 419 were similar (Table 1), the total biomass was higher only for the cultivar TME 419, suggesting that the carbon acquisition in TME 7 is not being fully translated into increases in biomass. This difference in biomass accumulation between cultivars, which is also apparent in field‐grown plants (Table S1), may reflect the importance of other components for cassava biomass production such as canopy structure (De Souza et al., 2017).

At *c*_i_ ≥500 μmol mol^−1^, *A*_sat_ appears limited by capacity for triose phosphate utilization (TPU limited) in three of the cultivars (Figure 2). Although the photosynthetic rates observed in these experiments are lower than those measured in other studies (El‐Sharkawy, 2016), the TPU limitation is evidenced here by a plateauing of the response to *A*_sat_ to *c*_i_ and a concomittant decline in *J*_PSII_ determined from modulated chlorophyll fluorescence with increasing *c*_i_ (Long & Bernacchi, 2003; Sharkey et al., 1986). The results suggest a capacity to utilize TPU was 20% and 26% above the observed *A*_sat_ in the two landraces (TME 7 and TME 419) at ambient [CO_2_], but only 4% higher in the bred TMS 98/0581. As under current atmospheric [CO_2_], the photosynthetic rates are largely Rubisco limited (Figure 2), our results do support the increase in photosynthesis observed under elevated [CO_2_] (Rosenthal et al., 2012) even considering this TPU limitation. Hence, while TPU limitation is not restrictive to photosynthesis under current conditions, and it might not be a problem with a slightly increase in [CO_2_] concentration, it will set a ceiling on improving photosynthetic efficiency by increasing efficiencies of Rubisco carboxylation and RubP regeneration as well as increased mesophyll conductance. The results suggest that understanding the basis of this TPU limitation will be critical to improving overall photosynthetic efficiency in this crop, at least during the critical crop establishment phase.

Capacity for triose phosphate utilization is defined by the plant's ability to convert triose phosphate into sucrose and starch. Thus, if sucrose or starch synthesis is reduced, the pool of triose phosphate increases, limiting the amount of inorganic phosphate (*Pi*) available for photophosphorylation (Sharkey, 1985). Consequently, TPU limitation of photosynthesis not only can be a reflection of a lack of sink for growth or storage but can also reflect inadequate capacity to produce starch and sucrose at the level of the leaf (Long & Bernacchi, 2003; Sharkey, Bernacchi, Farquhar, & Singsaas, 2007). When this occurs, it can cause a negative feedback on photosynthetic capacity (Yang, Preiser, Li, Weise, & Sharkey, 2016). Due to the large production of tuberous roots observed in cassava, the reduced sink strength at the crop level is not usually expected. However, establishment normally occurs prior to bulking of the tuberous roots. In addition, there are substantial differences in sink capacity among cassava genotypes (Gleadow, Evans, McCaffery, & Cavagnaro, 2009; Ihemere et al., 2006; Rosenthal et al., 2012). While V_TPU_ reported here for the landraces was close to the average recorded across several species of 10.1 μmol m^−2 ^s^−1^ at 25°C, it is low compared to other food crops, such as rice (14.5 μmol m^−2 ^s^−1^), rye (18.6 μmol m^−2^ s^−1^), and wheat (15.8 μmol m^−2 ^s^−1^) (Jaikumar, Snapp, & Sharkey, 2013; Wullschleger, 1993). For the bred farmer‐preferred cultivar, *V*_TPU_ was about one‐third of these values (Table 1). Since *V*_TPU_ sets the upper limit on the maximum *A*_sat_ that a leaf can achieve under any conditions, the results suggest a strong limitation on CO_2_ assimilation during the crop establishment phase compared to other food crops. Since *V*_TPU_ limitation feeds back on capacity in terms of both *V*_cmax_ and *J*_max_ (Yang et al., 2016), this may also explain the relatively low values for these parameters at this growth stage.

The lack of sink is usually associated with an increase in leaf starch content (Stitt, 1991). All the three cultivars that showed TPU limitation had more leaf starch at dusk (Figure 2; Table 2). However, this starch accumulation did not reduce the photosynthetic rates significantly during the afternoon (Figure 5) indicating that the amount of transitory starch accumulation observed in leaves of these three cultivars is not related to the observed TPU limitation. Furthermore, all cultivars were able to utilize the starch between dusk and dawn periods indicating that there is a sink that demands the degradation of starch during the night. Nevertheless, leaf TSS did not show a significant variation between dusk and dawn periods (Table 2), suggesting an impairment in the utilization of these sugars that can be related to a reduced capacity to transport them to sink tissues. It has been demonstrated that the starch synthesis in the tuberous roots is a limiting step in the cassava metabolism (Ihemere et al., 2006) and that the accumulation of starch in tuberous roots is positively correlated with the utilization of soluble sugars in leaves (Luo & Huang, 2011). However, at this growth stage, shoot development is the major sink suggesting that TPU limitation is more likely imposed at the leaf level, possibly through inadequate capacity for starch or/and sucrose. Based on the past studies, this is most likely imposed by inadequate activities of ADP‐glucose pyrophosphorylase, cytosolic fructose‐1.6‐bisphosphatase, or/and sucrose phosphate synthase (Yang et al., 2016), making these key targets for upregulation. TMS 30572, which did not show TPU limitation, utilized ~30% of the leaf TSS during the night (Table 2), and had, proportionally, more starch in its tuberous roots than the other cultivars (Figure S2).

Due to the fact that the plants evaluated in this study were young plants and were still at the beginning of tuberous roots development, we cannot discard the possibility that the TPU limitation observed be transitory. However, this limitation was detected even in TME 419, which produced 435% more tuberous root biomass than the other cultivars (Figure 6). Also, the reduced capacity of starch synthesis in the tuberous roots observed by Ihemere et al. (2006) was measured in 6‐month‐old plants. This suggests that even during later growth stages when the accumulation of biomass in tuberous roots is higher, sink limitation could affect photosynthetic capacity in cassava. Furthermore, this limitation could become more pronounced if photosynthetic rates are increased by genetic manipulation. However, the significantly higher capacity for TPU in the two landraces suggests some opportunity for breeding increased capacity (Figure 2; Table 1). This may appear at odds with the finding of Rosenthal et al. (2012) who found a very strong stimulation of yield when cassava photosynthesis was stimulated by elevated CO_2_ under open‐air concentration enrichment. However, the plants were grown to a significant size in a common greenhouse environment, before being transferred to the field treatment plots. That is, this earlier experiment did not analyze the early establishment phase, examined in the present study.

Of the four cultivars analyzed, it is curious that photosynthetic capacity and iWUE were under most conditions highest in the landraces. An overriding factor in genetic improvement of cassava has been disease resistance. For example, TMS 98/0581 was bred for its resistance to cassava mosaic virus disease (CMD) and was shown to be the most resistant of 40 different cultivars surveyed in 2007, while TME 419 showed only moderate resistance in the same study (Egesi, Ogbe, Akoroda, Ilona, & Dixon, 2007). The older improved cultivar TMS 30572 was bred for resistance to CMD, cassava bacterial blight, cassava anthracnose disease, cassava mealybug, and cassava green mite (Eke‐Okoro & Njoku, 2012), although its resistance to CMD has now broken down (Egesi et al., 2007). Clearly with a crop vulnerable to such devastating diseases and pests, overcoming these has been paramount. However, from the very limited sample used in this study, it appears that photosynthetic capacity and water use efficiency could have declined with the focus on selection for pest and disease resistance. Even though the limitation in genetic diversity of this study do not allow further extrapolations to breeding programs, the results suggest that with now off the shelf equipment for rapid and nondestructive measurement of photosynthetic capacity, it would be possible to control for loss of photosynthetic capacity in selecting for improved pest and disease resistance (Long & Bernacchi, 2003; Stinziano et al., 2017). While limited, the finding here suggests that a wider range of African cultivars should be screened to establish whether this is a pervasive change. Bioengineering would allow substantial increases in photosynthetic capacity and have the advantage in a clonal crop that it could transfer increased capacity into elite cultivars with high pest and disease resistance, without the need for backcrossing (Kromdijk & Long, 2016; Kromdijk et al., 2016; Long et al., 2015). In theory, the use of bioengineering would be a far more rapid means of increasing photosynthetic capacity, since it would avoid the many rounds of backcrossing required in conventional breeding. This though requires an effective and efficient system for genetic transformation of this crop and a regulatory framework for release of such material in the countries in which cassava is most important as a food source. Clearly, this would need to be coupled with increased sink capacity, at least in the all‐important establishment phase.

## CONFLICT OF INTEREST

None declared.
