# Supplementary material for: Toward improving photosynthesis in cassava: Characterizing photosynthetic limitations in four current African cultivars
Source: Food Energy Secur. 2018 Apr 16;7(2):e00130. doi: 10.1002/fes3.130 (PMC6049889; doi:10.1002/fes3.130)
Supplement: Supplementary file 1 [file FES3-7-na-s001.docx]

**SUPPORTING INFORMATION FOR**

**TOWARD IMPROVING PHOTOSYNTHESIS IN CASSAVA: CHARACTERIZING PHOTOSYNTHETIC LIMITATIONS IN FOUR CURRENT AFRICAN CULTIVARS**

Amanda P. De Souza & Stephen P. Long

Content:

Figures S1-S3

Table S1

**
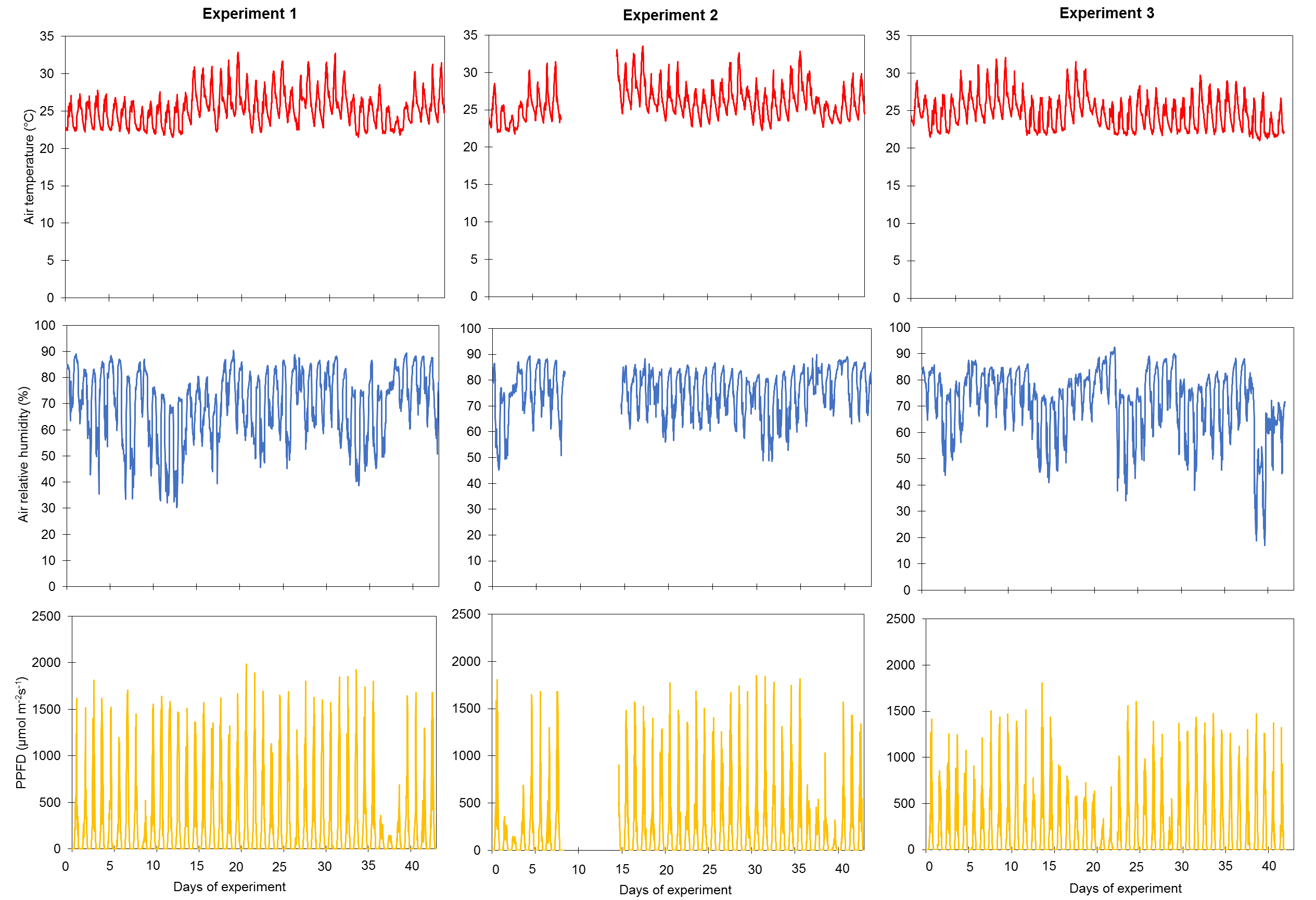
**

**Fig. S1.** Air temperature, air relative humidity and photosynthetic photon flux density (PPFD) from the three experiments with cassava during summer 2016. Gap (6 days) during the experiment 2 shows the period that data logger did not log the data due to technical problems and maintenance. Experiment 1 = May 27 to July 13; Experiment 2 = July 01 to August 18; Experiment 3 = August 19 and September 29.

**
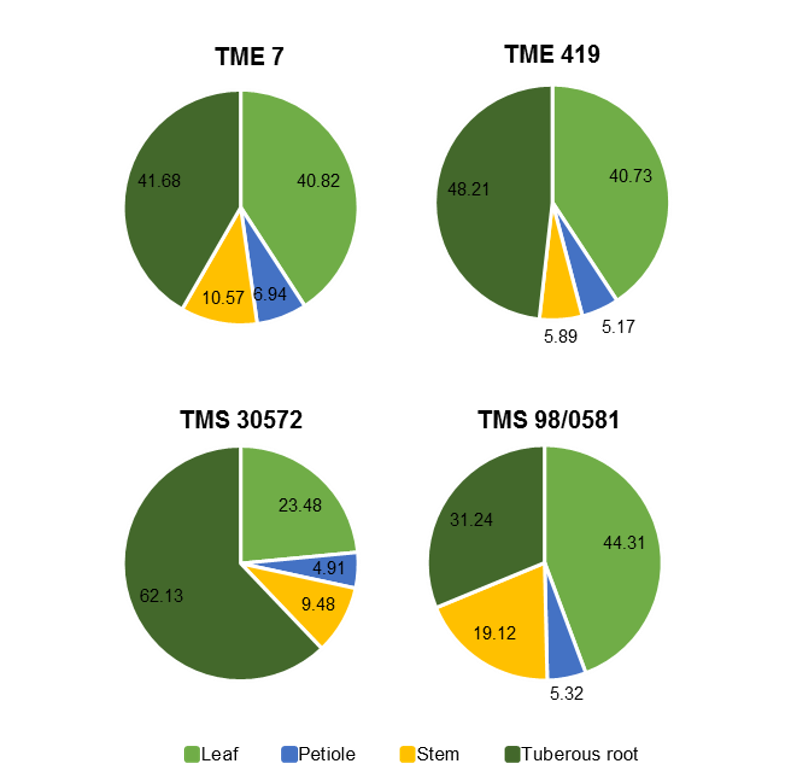
**

**Fig. S2.** Distribution of starch in different organs of cassava in the four cultivars (TME 7, TME 419, TMS 30572 and TMS 98/0581). Values are given as the percentage of the total plant starch content.


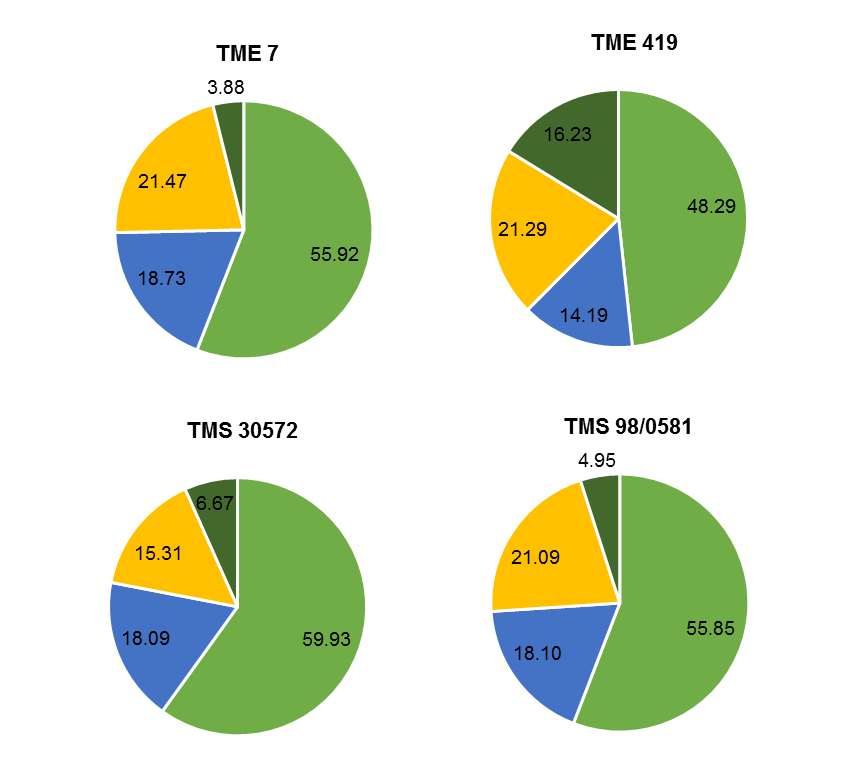

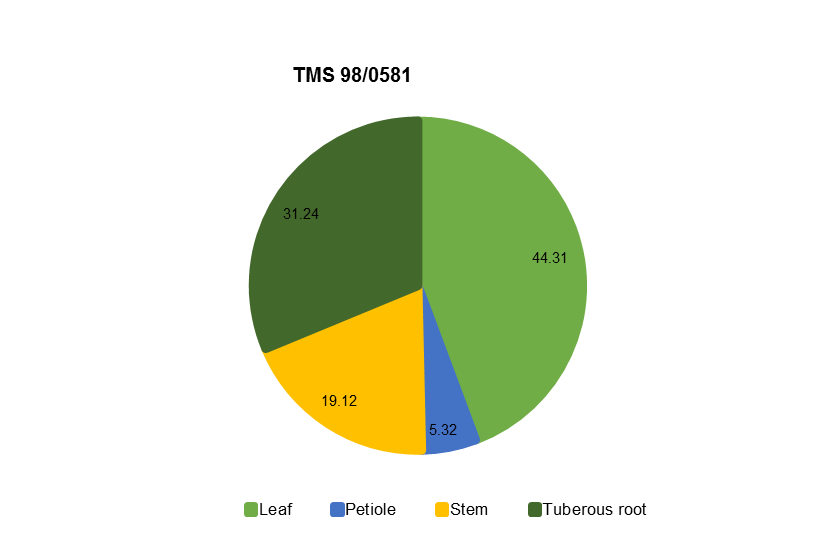


**Fig. S3.** Biomass partition among the different organs of cassava in the four cultivars (TME 7, TME 419, TMS 30572 and TMS 98/0581). Values are given as the percentage of the total biomass.

**Table S1**. Fresh root biomass (kg), fresh shoot biomass (kg), and harvest index in field experiments of the four cultivars used in this study. Values are average ± SD. Data obtained from cassava database, available at https://cassavabase.org/.

| **Cultivar** | **Fresh root biomass** | **Fresh shoot biomass** | **Harvest index** |
| --- | --- | --- | --- |
| TMS 98/0581 | 22.1 ± 14.8 | 20.3 ± 23.3 | 0.54 ± 0.12 |
| TMS 30572 | 27.5 ± 27.8 | 12.5 ± 12.6 | 0.60 ± 0.60 |
| TME 419 | 24.5 ± 28 | 11.4 ± 5.5 | 0.59 ± 0.13 |
| TME 7 | 10.5 ± 9.5 | 7.9 ± 6.1 | 0.51 ± 0.18 |
